# Free radical scavenging and anti-isolated human LDL oxidation activities of *Butea superba* Roxb. extract

**DOI:** 10.1186/s12906-024-04373-w

**Published:** 2024-02-03

**Authors:** Kittipot Sirichaiwetchakoon, Griangsak Eumkeb

**Affiliations:** School of Preclinical Sciences, Institute of Science, Suranaree University of Technology, 111 University Avenue, Suranaree Subdistrict, Nakhon Ratchasima, 30000 Muang District Thailand

**Keywords:** *Butea superba* Roxb., Anti-LDL oxidation, Antioxidation, And atherosclerosis

## Abstract

**Background:**

*Butea superba* Roxb. (*B. superba*), is an herbal plant traditionally used for rejuvenation. Additionally, there have been reports on its antioxidant properties. Low-density lipoproteins (LDL) oxidation is the leading cause of cardiovascular diseases (CVDs). Natural products with antioxidant properties have the potential to inhibit LDL oxidation. However, no work has been done about the anti-isolated human LDL oxidation of *B. superba* extract (BSE). This study aimed to investigate the antioxidant potential of BSE and its ability to prevent isolated human (LDL) oxidation induced by free radical agents.

**Methods:**

The antioxidant properties were investigated by antioxidant assays, including 2,2-diphenyl-1-picrylhydrazyl (DPPH), 2,2-azinobis-(3-ethylbenzothiazoline)-6-sulfonic acid (ABTS), ferric reducing ability power (FRAP), nitric oxide (NO) and peroxynitrite scavenging assay. More so, anti-isolated human LDL oxidation activities were evaluated by 2,2'-azobis (2-amidinopropane) dihydrochloride (AAPH) and 3-morpholinosydnonimine hydrochloride (SIN-1) induced LDL oxidation assay.

**Results:**

BSE exhibited a significant (*p* < 0.05) antioxidant activity in all the test systems, demonstrating its potential as a potent free radical scavenger. It displayed scavenging effects on DPPH (*p* < 0.05; IC_50_ = 487.67 ± 21.94 µg/ml), ABTS (*p* < 0.05; IC_50_ = 30.83 ± 1.29 µg/ml). Furthermore, it generated significantly (*p* < 0.05) increased antioxidant capacity in a dose-dependent manner in FRAP assay and exhibited significantly (*p* < 0.01) higher percent NO scavenging activity than gallic acid. Besides, BSE at 62.5 µg/ml exhibited a considerable percent peroxynitrite scavenging of 71.40 ± 6.59% after a 2 h period. Moreover, BSE demonstrated anti-isolated human LDL oxidation activity induced by AAPH and SIN-1 (*p* < 0.05) and revealed scavenging activity similar to ascorbic acid (*p* > 0.05). Identifying the main constituents of BSE revealed the presence of genistein, daidzein, and biochanin A through Liquid Chromatography-Mass Spectrometer/Mass Spectrometer (LC–MS/MS) analysis.

**Conclusion:**

This is the first report that the presence of isoflavones in BSE could play an important role in its antioxidation and isolated human LDL oxidation scavenging properties. These findings suggest the potential for developing antioxidant herbal supplements. However, further studies must be investigated, including efficacious and safe human dosages.

## Background

Over the past decade, herbal supplements or alternative medicines have become increasingly popular [1]. This increasing trend can be attributed to several factors, including the potential for fewer adverse effects compared to synthetic drugs. Additionally, traditional medicinal plants used in these supplements are often more affordable and easily consumable [2].

A free radical is characterized as a molecular species capable of existing independently, possessing an unpaired electron in an atomic orbital. These unpaired electrons make the free radical highly reactive and unstable. This unpaired electron can lead to a chain reaction, generating new free radicals and causing cellular damage [3]. Free radicals and other oxidants have become increasingly significant in biology and medicine [4]. The close association between oxidative stress and lifestyle-related diseases has become well known. Oxidative stress is defined as a “state in which oxidation exceeds the antioxidant systems in the body secondary to a loss of the balance between them [5]. Cardiovascular diseases (CVDs) are one of the leading causes of death worldwide [6], and the oxidation of low-density lipoprotein (LDL) has been identified as a key event in the development of atherosclerosis, which is a major risk factor for CVDs [7]. Therefore, the search for natural products with antioxidant properties capable of preventing LDL oxidation has received much attention in recent years. LDL oxidation is a complex process involving generating reactive oxygen species (ROS) and reactive nitrogen species (RNS), which can lead to the modification of LDL particles, making them more susceptible to macrophage uptake and the subsequent formation of foam cells. This process is a critical step in developing atherosclerosis, characterized by forming plaques in arterial walls [8–10]. Atherosclerosis is considered one of the main causes of cardiovascular diseases, which remain a leading cause of morbidity and mortality worldwide [11].

Herbal remedies for managing ROS and RNS are an interesting area of research within natural medicine and traditional healing practices. Some herbals and natural compounds have shown potential in helping to mitigate the effects of ROS and RNS [12, 13]. Considering the essential role of LDL oxidation in atherosclerosis development, finding natural compounds with antioxidative properties could have significant implications for preventing the progression of atherosclerosis and cardiovascular diseases.

*B. superba*, a plant in the family Leguminosae, has been traditionally used for its various pharmacological properties, such as rejuvenation, improving sexual performance, and preventing erectile dysfunction [14]. Moreover, several studies have reported the antioxidant potential of *B. superba* [15, 16]. Therefore, *B. superba* has the potential to develop into a health product for LDL oxidation prevention. For these reasons, *B. superba* could provide a potential natural dietary supplement or functional food for promoting overall health and preventing oxidative stress-related diseases.

Although some biological activities of *B. superba* have been reported, no work has been done about the anti-isolated human LDL oxidation. Therefore, this study aimed to investigate the free radical scavenging potential of *B. superba* extract as a free radical scavenger and inhibitor of isolated human LDL oxidation by AAPH or SIN-1. The investigation could contribute to its protective effects against atherosclerosis and cardiovascular diseases.

## Methods

### Plant material

Fresh tuberous roots of *B. superba* were collected from Chiang Rai province, Thailand, and authenticated by Dr. Paul J Grote, a lecturer and plant biologist at the Institute of Science, Suranaree University of Technology. The plant specimen was compared to the voucher specimen (BCU 1046) and deposited at Forest Herbarium, National Park, Wildlife, Plant Conservation Department, Ministry of Natural Resources and Environment, Thailand.

### Plant extraction process

The ethanolic extraction of *B. superba* was carried out according to the methods described by Eumkeb et al. and Sirichaiwetchakoon et al. with minor modifications [14, 17, 18]. The plant extraction process was started by washing, grinding, and drying in an oven at 60 ºC for 72 h. The resulting dried powdered tuber roots of *B. superba* weighing 2 kg were subjected to continuous extraction using a Soxhlet apparatus with ethanol for 12 h. Subsequently, the extract was filtered through Whatman No. 1 filter paper. The ethanolic extract was evaporated under reduced pressure at 50 °C and lyophilized to obtain the powder of BSE (30.5 g). The BSE was stored at -20 °C until used. The extract's percentage yield was found to be 1.525% w/w, which could be calculated using the following formula;

$$\text{percent yield of the extract}=\frac{\text{weight of the dry extract}}{\text{weight of the dry plant}}\times {100}$$

### Chemicals and reagents

Genistein (product number; G6649), daidzein (product number; 16,587), biochanin A (product number; 92,142), 2,2-diphenyl-1-picrylhydrazyl (DPPH) (product number; D9132), 2,2-azinobis-(3-ethylbenzothiazoline)-6-sulfonic acid (ABTS) (product number; A3219), sulfanilamide (product number; S9251), naphthylethylenediamine dihydrochloride (NED) (product number; N9125), 3-morpholinosydnonimine hydrochloride (SIN-1) (product number; M5793), 2,4,6-tris(2-pyridyl)-s-triazine (TPTZ) (product number; 93,285), diethylene-triamine-pentaacetic acid (DTPA) (product number; D1133), Evans blue (product number; E2129), 2,2′-azobis (2-methylpropionamidine) dihydrochloride (AAPH) (product number; 440,914), trichloroacetic acid (TCA) (product number; T6399), 2-thiobarbituric acid (product number; T5500), low density from human plasma (product number; L8292), gallic acid (product number; G7384), ascorbic acid (product number; 1,043,003), potassium persulfate (product number; 216,224), ammonium acetate (product number; A1542), phosphate-buffered saline (PBS) (product number; P3619), and ferric chloride (product number; 451,649) were purchased from Sigma-Aldrich Chemical (St. Louis, MO, USA). Other reagents used were analytical grade.

### LC–MS/MS instrument and conditions

The LC–MS/MS technique was employed to identify the chemical constituents present in *B. superba*. The combination of chromatographic separation in the LC–MS/MS system was combined from an Agilent HPLC 1290 Infinity with a mass analyzer 6490 Triple Quad LC/MS from Agilent Technologies, equipped with an electrospray ionization (ESI) source system. The setup consisted of an auto-sampler, a binary pump, and a vacuum degasser. An Agilent ZORBAX Rapid Resolution High Definition (RRHD) SB-C18, 2.1 mm id × 150 mm (1.8 µm) was used for chromatographic separation. The mobile phase system involved two solvents: solvent A, comprising 13 mM ammonium acetate buffer adjusted to pH 4 with 0.1% acetic acid, and solvent B, consisting of 0.1% acetic acid in methanol. The gradient elution method employed a ratio of solvent A to solvent B starting at 100:0, which gradually shifted to 60% solvent B at 5 min and reached 100% solvent B at 30 min, operating at a flow rate of 0.25 ml/min. The sample injection volume was set at 20 µl, and the column temperature was maintained at 40 °C throughout the analysis. Genistein, daidzein, and biochanin A were used as standard.

### DPPH scavenging activity

The DPPH scavenging activity of BSE was assessed using a method described by Brand-Williams et al. [19] with little modifications. In brief, BSE and gallic acid with concentrations ranging from 0 to 1,000 µg/ml were prepared using 0.002% DPPH reagent in methanol. PBS was used as a negative control. These extract solutions were thoroughly mixed and then incubated in the dark at room temperature for 30 min. The reactions were performed in a 96-well plate with gentle shaking. Subsequently, the absorbance of all samples was measured at 515 nm using a spectrophotometer. The procedure for assessing the radical scavenging activity was repeated three times, and the percentage of DPPH scavenging was calculated using the following formula;

$$\text{percent DPPH }\text{s}\text{cavenging}=\text{(1}\,-\frac{{\text{OD}}_\text{sample}-{\text{OD}}_\text{sample blank}}{{\text{OD}}_\text{control}-{\text{OD}}_\text{sample blank}}{)\times 100}$$

### ABTS scavenging activity

The ABTS scavenging activity of BSE and ascorbic acid was investigated following the method of Re et al. [20] with slight modifications. To generate the ABTS^+^ radical cation, a mixture of 7 mM ABTS stock solution and 2.45 mM potassium persulfate (final concentration) was prepared and kept in the dark at room temperature for 16 h until the reaction was completed. The absorbance of the solution was adjusted to 0.70 (± 0.02) by diluting it with ethanol at room temperature before initiating the reaction. In a 96-well plate, 90 μl of the ABTS^+^ radical cation solution was mixed and shaken with 10 μl of the test sample at various concentrations (0–37.5 μg/ml final concentration) for 45 s. After 15 min of the reaction, the absorbance was measured at 734 nm using a spectrophotometer. The assay was repeated three times, and the percentage inhibition of absorbance was described as an ABTS scavenging percentage according to the following equation;

$$\text{percent ABTS }\text{s}\text{cavenging}=\text{(1}\,-\frac{{\text{OD}}_\text{sample}-{\text{OD}}_{\text{sa}\text{mple blank}}}{{\text{OD}}_\text{control}-{\text{OD}}_\text{sample blank}}{)\times 100}$$

### FRAP activity

The FRAP assay was conducted according to the method described by Benzie et al. [21] with slight modifications. This assay is based on the ability of antioxidants to reduce a ferric-tripyridyltriazine complex to its ferrous, colored form in the presence of antioxidants. In brief, the working FRAP reagent was composed of 300 mM acetate buffer (pH 3.6), a solution containing 10 mM TPTZ in 10 mM hydrochloric acid, and 20 mM ferric chloride, mixed in a ratio of 10:1:1 (v/v/v). To measure the antioxidant capacity of BSE, samples were prepared in various concentrations of the FRAP reagent, ranging from 0 to 1000 µg/ml. The mixture was incubated in a 96-well plate for 6 min. The absorbance at 595 nm was measured using a microplate reader. FRAP values were calculated as milligrams of Trolox equivalent antioxidant capacity (TEAC) per gram of dry extract, providing a quantitative measure of the antioxidant capacity of the BSE samples. The experiment was repeated three times.

### Nitric oxide scavenging activity

The nitric oxide scavenging assay was performed following the method of Yen et al. [22] with little modifications. In this experiment, SIN-1 was used to generate RNS. One of the end-products of the reaction involving RNS is nitrite. If BSE could scavenge NO, the formation of nitrite would be reduced. The concentration of nitrite was quantified using the Griess reaction assay. The sample solution in a 96-well plate was prepared by mixing 0.25 mM SIN-1 with PBS (pH 7.4) and BSE or gallic acid at various doses (0–250 μg/ml final concentration), in a final volume of 40 μl. Samples were then incubated at 25 °C for 30 min. Afterward, 80 μl of 0.33% sulfanilamide in 20% glacial acetic acid was added to the incubated samples and shaken for 5 min. Subsequently, 80 μl of 0.1%NED was added, and the final samples were incubated at 25 °C for 15 min. Nitric oxide scavenging was measured spectrophotometrically at 540 nm against a blank sample. The assay was repeated three times, and the percentage of nitric oxide radical scavenging was calculated and expressed using the formula as follows;

$$\text{percent Nitric oxide radical}\,\text{scavenging}=\text{(1}\,-\frac{{\text{OD}}_\text{sample}-{\text{OD}}_\text{sample blank}}{{\text{OD}}_\text{control}-{\text{OD}}_\text{sample blank}}{)\times 100}$$

### Peroxynitrite scavenging activity

SIN-1 was used as a peroxynitrite donor in this assay, and the peroxynitrite scavenging activity was investigated using an Evans blue bleaching assay [23]. The assay was performed on a 96-well plate. The reaction mixture consisted of 50 mM phosphate buffer (pH 7.4), 0.1 mM DTPA, 90 mM NaCl, 5 mM KCl, 12.5 μM Evans Blue, 1 mM SIN-1, and 62.5 μg/ml of BSE and gallic acid, in a final volume of 200 μl. The mixture was incubated at 37 °C for 180 min, and the absorbance was measured at 611 nm every 30 min. The assay was repeated three times. The percentage scavenging of peroxynitrite at different time points was calculated by comparing the absorbance of samples with the blank and expressed as the percent optical density of Evans Blue by the following equation;

$$\text{percent optical density of Evans Blue }=\text{(1}\,-\,\frac{{\text{OD}}_{\text{sample b}\text{lank}}-{\text{OD}}_\text{sample}}{{\text{OD}}_{\text{sample b}\text{lank}}-{\text{OD}}_\text{control}}{)\times 100}$$

### AAPH-induced LDL oxidation activity

Before using human LDL for this assay, this experiment was approved by the ethics committee of Suranaree University of Technology (EC-66–0019). The AAPH-induced LDL oxidation assay was evaluated following the method of Barcelos et al. [24] with modifications. AAPH is a ROS generator that produces ROS upon activation. To prepare the sample solution, AAPH at a concentration of 20 mM was mixed with 250 µg/ml of LDL cholesterol and 100 µg/ml of BSE or ascorbic acid added to the mixture. The control sample consisted of 250 µg/ml of LDL cholesterol mixed with PBS. In contrast, the AAPH-treated sample contained 250 µg/ml of LDL cholesterol combined with AAPH at a concentration of 20 mM. All samples were incubated at 37 °C, collected at various time points (0, 30, 60, 90, 120, 150, 180 min), and placed at -20 °C before stopping the reaction. Plasma oxidation product was measured by Thiobarbituric acid reacting substances (TBARS). For TBARS determination, collected samples at different time points were mixed with 20%TCA to precipitate protein. The samples were then centrifuged at 10,000 rpm at 4 °C for 10 min. The supernatant was collected and mixed with 1% thiobarbituric acid. The samples were then heated at 95 °C for 20 min. TBARS concentration was determined by measuring UV absorption at 532 nm and comparing it with a malondialdehyde (MDA) standard curve. The results were expressed as MDA equivalence. The assay was performed in triplicate.

### SIN-1-induced LDL oxidation activity

To investigate the effect of RNS-mediated LDL oxidation, SIN-1 was used as a peroxynitrite donor. The assay was performed using a previous method [20] with few modifications. The sample solution consisted of 1 mM of SIN-1 challenged with 250 µg/ml of LDL cholesterol and mixed with 100 µg/ml of BSE or ascorbic acid. The control sample was 50 µg/ml of LDL cholesterol mixed with PBS. The SIN-1-treated LDL sample contained 250 µg/ml of LDL cholesterol mixed with 1 mM of SIN-1. The samples were then incubated at 37 °C for 18 h and subsequently stored at -20 °C to stop the reaction. Plasma oxidation products were measured using the TBARS assay, and the results were expressed as MDA equivalence. The assay was repeated three times.

### Statistical analysis

All data were expressed as mean ± standard error of the mean (SEM.). Statistical analyses were analyzed using SPSS version 18.0. The significant differences between groups in the DPPH, ABTS, and NO scavenging assays were analyzed by independent t-test. One-way analysis of variance (ANOVA) with a Tukey's HSD post-hoc test was used to compare the FRAP, Peroxynitrite, AAPH, and SIN-1 scavenging assays. Statistical significance was considered at *p* < 0.05, and the presented data were derived from a minimum of three independent experiments.

## Results

### Chemical identification of BSE by LC–MS/MS

**Fig. 1:**
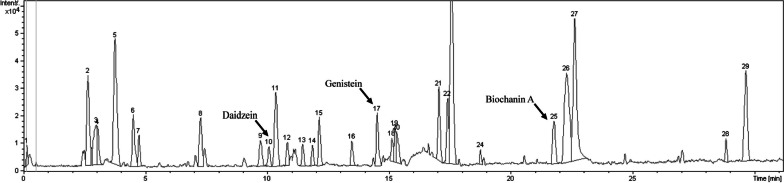
LC–MS/MS Chromatogram of compounds in BSE

The LC–MS/MS analysis (Fig. 1) revealed that daidzein, genistein, and biochanin A were detected in the BSE. Moreover, the quantification of the analytes was performed. The result expressed that BSE at 1000 µg/ml contained daidzein, genistein, and biochanin A at 15.83 µg/ml,14.97 µg/ml, and 39.1 µg/ml, respectively.

### DPPH scavenging activity

**Fig. 2:**
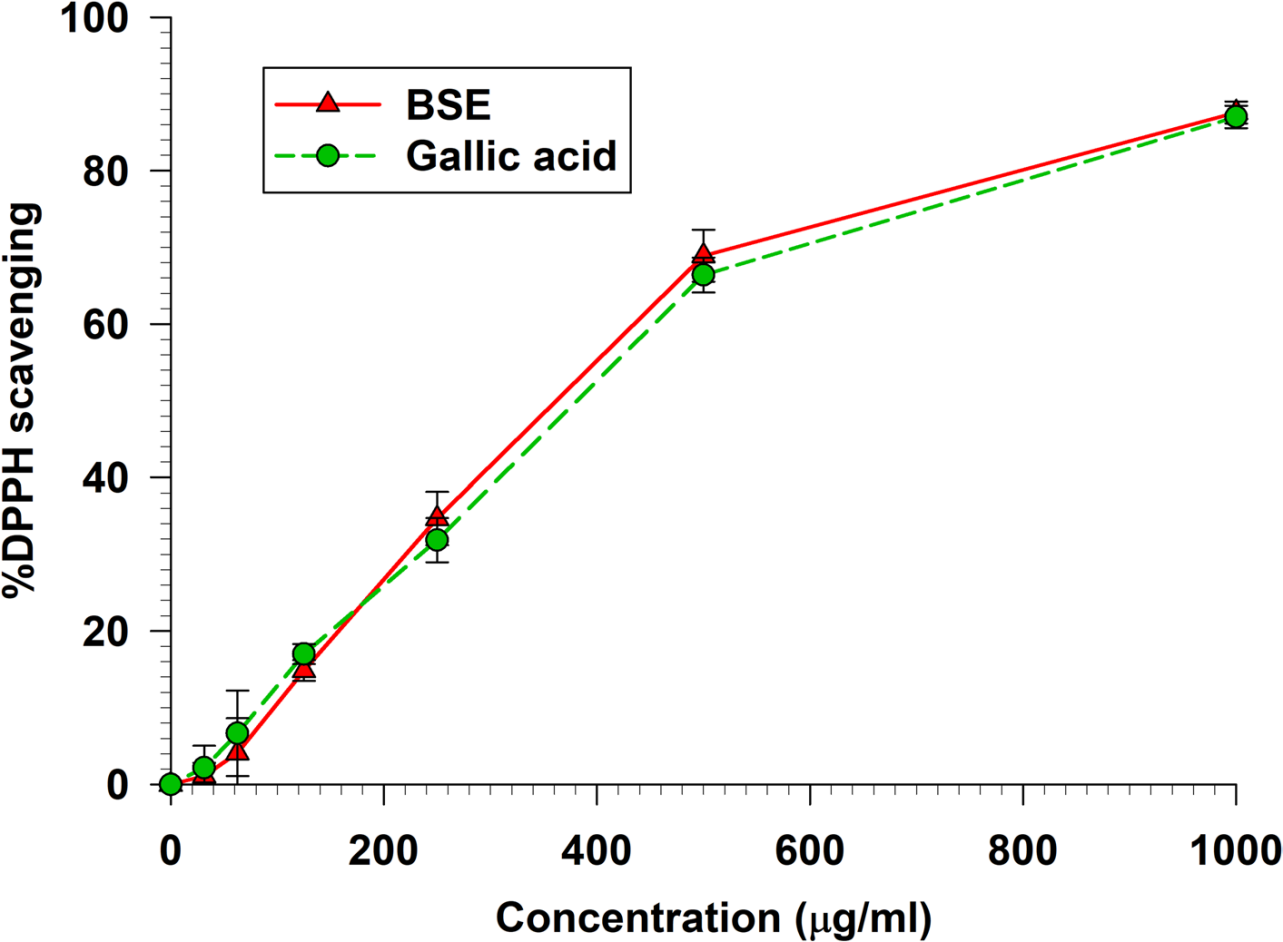
The DPPH radical scavenging activity of BSE and gallic acid

The DPPH radical scavenging assay is a widely used method in biochemistry and analytical chemistry to measure the antioxidant activity of a substance. The antioxidant capacity of BSE was investigated compared to gallic acid, which occupies potent antioxidant and free radical scavenging activities. Our findings revealed that BSE exhibited the same antioxidant capacity compared to gallic acid within the concentration range of 0 µg/ml to 1000 µg/ml (*p* > 0.05) (Fig. 2). Moreover, the IC_50_ value of BSE was calculated at 487.67 ± 21.94 µg/ml and gallic acid at 492.8 ± 23.39 µg/ml (Table 1).

**Table 1:** The summary of scavenging activities of BSE and standard compound

Sample	Antioxidation assays					
	**DPPH (IC**_**50**_**)** **µg/ml**	**ABTS (IC**_**50**_**)** **µg/ml**	**NO (IC**_**50**_**)** **µg/ml**	**Peroxynitrite (percent optical density of evens blue at 3 h)**	**LDL oxidation** **(AAPH) (MDA equivalence** **at 3 h) nmole/mgProtein**	**LDL oxidation** **(SIN-1) (MDA equivalence)** **nmole/mgProtein**
BSE	487.67 ± 21.94	30.83 ± 1.29	85.17 ± 7.27	71.62 ± 5.46	4.15 ± 0.14	2.89 ± 0.45
Gallic acid	492.8 ± 23.39	-	236.38 ± 14.92	70.92 ± 2.24	-	-
Ascorbic acid	-	21.73 ± 0.30	-	-	3.91 ± 0.09	2.34 ± 0.10

Values are mean ± SEM (*n* = 3) and represent three independent experiments with similar results

Data represent the percentage of DPPH inhibition. Each value represents mean ± SEM (*n* = 3) and is representative of three independent experiments with similar results.

### ABTS scavenging activity

**Fig. 3:**
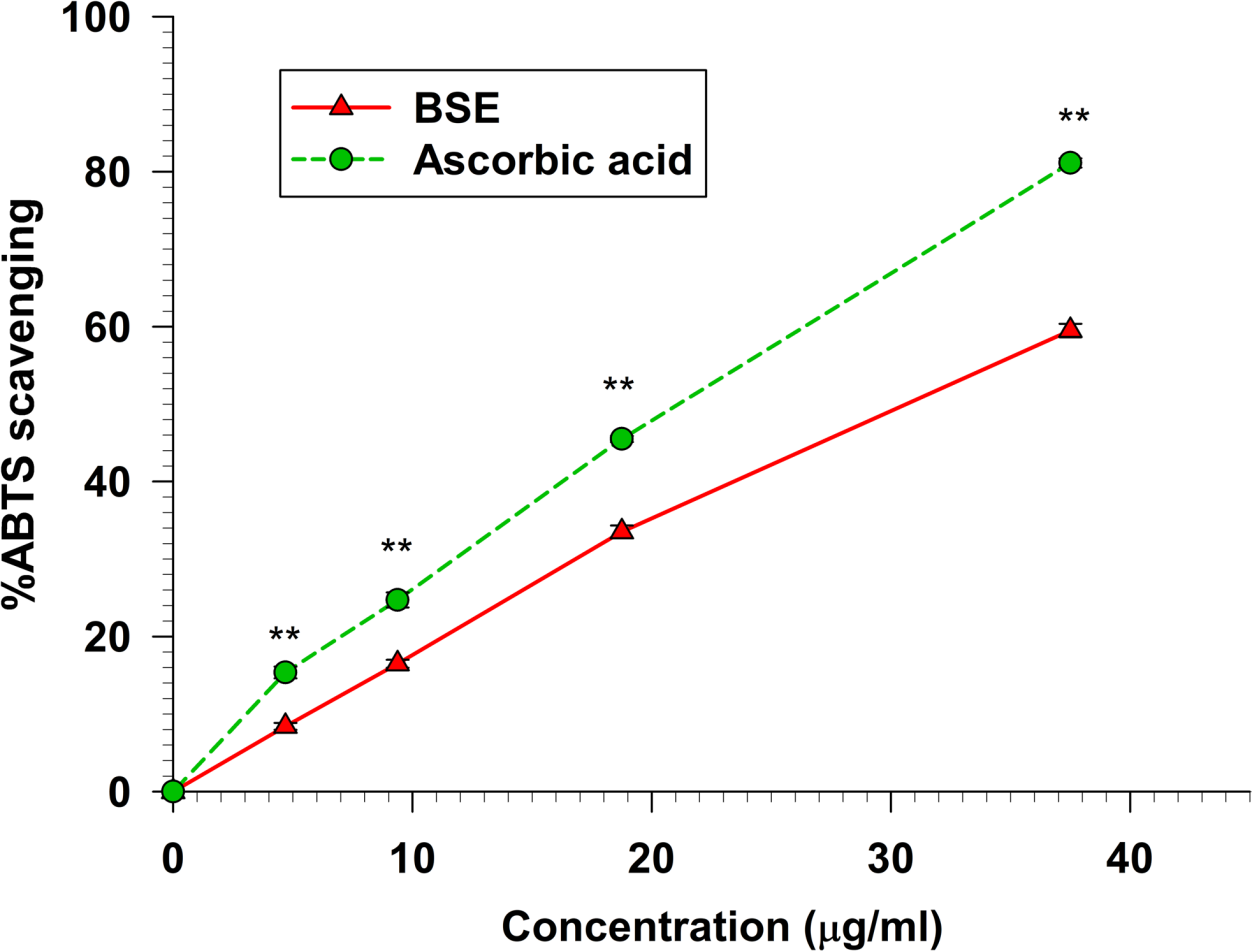
The ABTS radical scavenging activity of BSE and ascorbic acid

The ABTS scavenging assay was employed to assess the ability of the extract to neutralize the ABTS^+^ radical. Figure 3 illustrates the percent ABTS radical scavenging of BSE compared to ascorbic acid. Ascorbic acid exhibited significantly (*p* < 0.01) more potent inhibition of the ABTS^+^ radical than BSE within the concentration range of 0–37.5 µg/ml. The IC_50_ of BSE and Ascorbic acid were 30.83 ± 1.29 and 21.73 ± 0.30 µg/ml, respectively (Table 1).

Data represent the percentage of ABTS^+^ inhibition. Each value represents mean ± SEM (*n* = 3) and is representative of three independent experiments with similar results.

** indicates a significant difference between groups at *p* < 0.01.

### FRAP activity

**Fig. 4:**
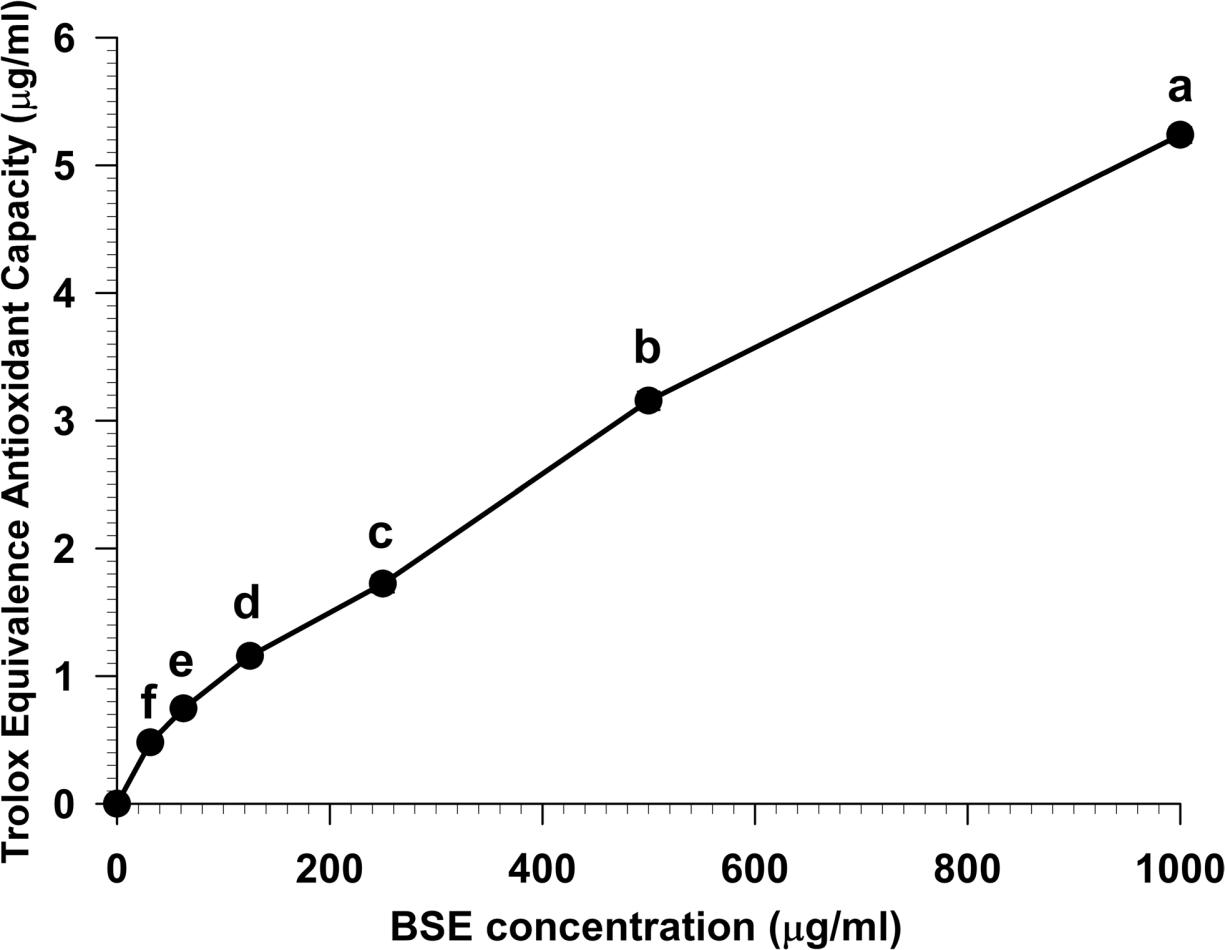
The FRAP assay of BSE

The ferric-reducing antioxidant power (FRAP) assay was performed to determine the antioxidant capacity of the extracts. This assay measured the ability of BSE to reduce ferric ions (Fe^3+^) to ferrous ions (Fe^2+^) in a redox-linked colorimetric reaction. The result showed that BSE had a potent ability to donate electrons and reduce Fe^3+^ to Fe^2+^, which was expressed as trolox equivalence antioxidant capacity (TEAC) (Fig. 4). At concentrations ranging from 0–1000 µg/ml, BSE generated a significant (*p* < 0.05) increase antioxidant capacity in a dose-dependent manner.

Data represent the trolox equivalence antioxidant capacity. Each value represents means ± SEM (*n* = 3) and is representative of three independent experiments with similar results. The significant difference in antioxidant capacity between concentrations, indicated by different superscript letters (a-f), was compared using ANOVA and Tukey’s HSD post hoc test at *p* < 0.05.

### Nitric oxide scavenging activity

**Fig. 5:**
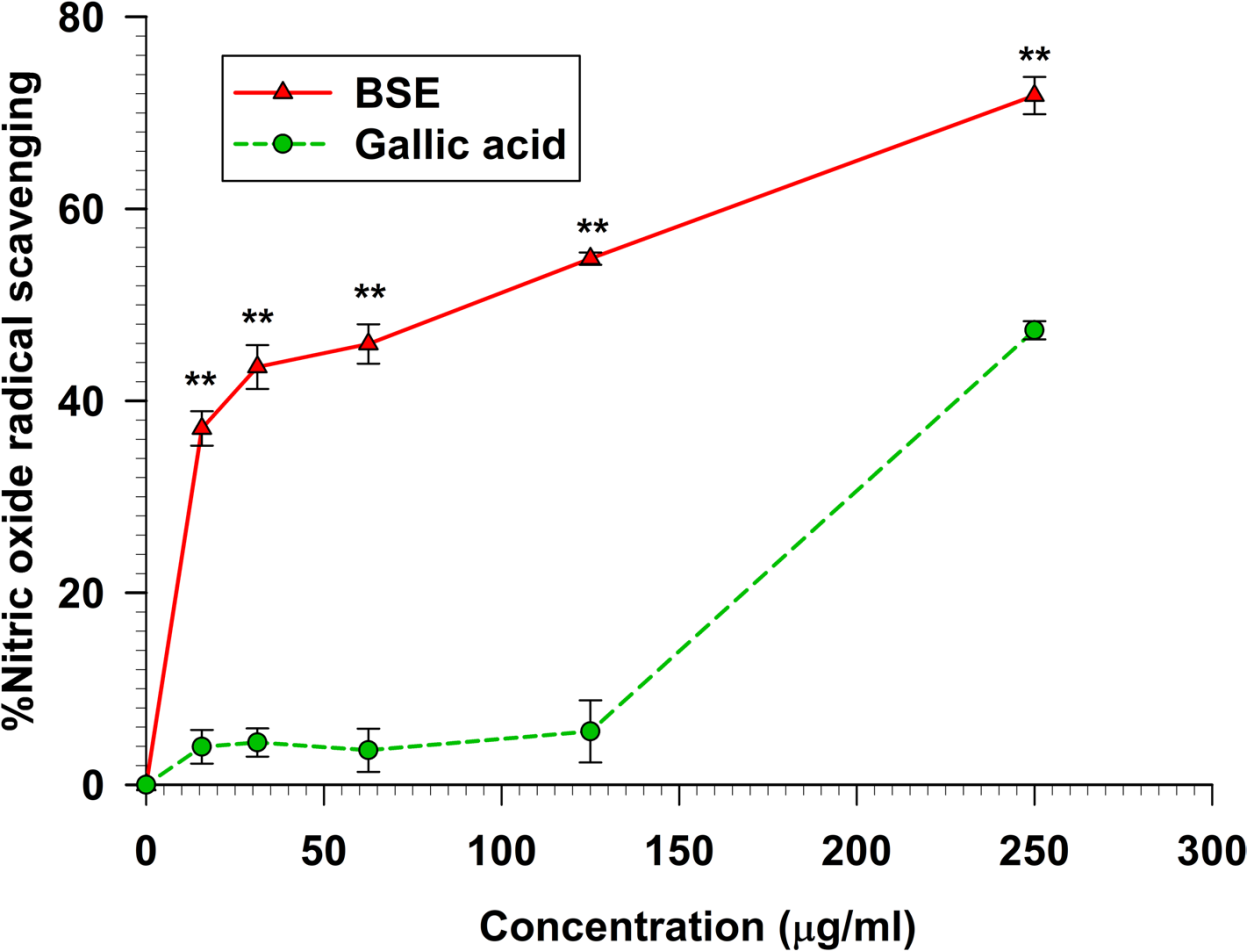
The nitric oxide radical scavenging activity of BSE and gallic acid

The nitric oxide scavenging assay is a method used to assess the ability of substances to neutralize or scavenge nitric oxide, a molecule involved in various physiological processes. The presence of nitric oxide scavenging activity was investigated by measuring the nitrite concentration using the Griess reaction method [22]. Figure 5 displays the percent inhibition of nitric oxide of BSE. Interestingly, the result demonstrated that BSE exhibited significantly (*p* < 0.01) higher percent nitric oxide scavenging activity than gallic acid at concentrations ranging from 0–250 µg/ml. The IC_50_ of BSE was 85.17 ± 7.27 µg/ml, indicating a more potent nitric oxide scavenging capability than gallic acid, whose IC_50_ was 236.38 ± 14.92 µg/ml (Table 1).

Data represent the percentage of nitric oxide inhibition. Each value represents mean ± SEM (*n* = 3) and is representative of three independent experiments with similar results. ** indicates a significant difference between groups at *p* < 0.01.

### Peroxynitrite scavenging activity

**Fig. 6:**
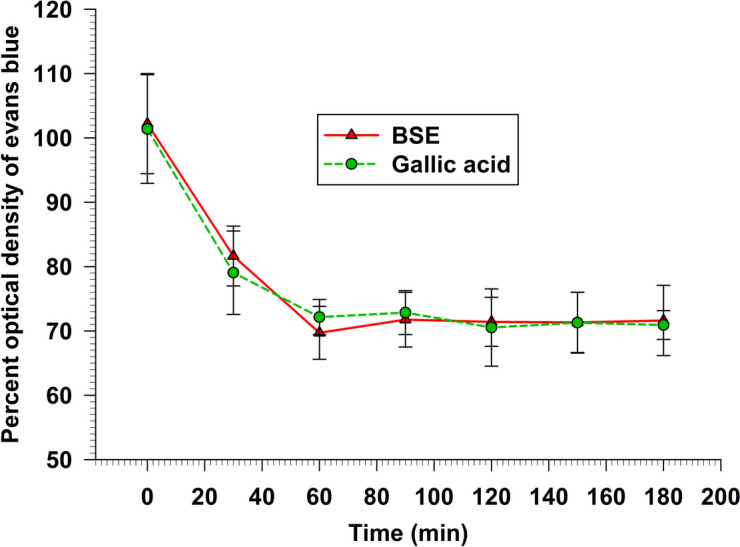
The peroxynitrite radical scavenging activity of BSE and gallic acid

The peroxynitrite scavenging assay is a method used to assess the ability of substances to neutralize or scavenge peroxynitrite, a highly reactive radical involved in oxidative stress. It is implicated in various pathological conditions, including inflammation, neurodegenerative disorders, and cardiovascular diseases. The Evan's blue assay was used to evaluate the peroxynitrite scavenging capacity, which was generated using SIN-1. The results are presented in Fig. 6 and Table 1. BSE concentration at 62.5 µg/ml exhibited a considerable percent peroxynitrite scavenging of 67.71 ± 7.12% after 1 h and plateau the scavenging activity over 3 h period. On the other hand, when compared to gallic acid, BSE displayed the same scavenging activity as gallic acid at various times (*p* > 0.05).

Data represent the percentage of the optical density of Evans blue at 611 nm at various times over 3 h. Each value represents mean ± SEM (*n* = 3) and is representative of three independent experiments with similar results.

### AAPH-induced LDL oxidation activity

**Fig. 7:**
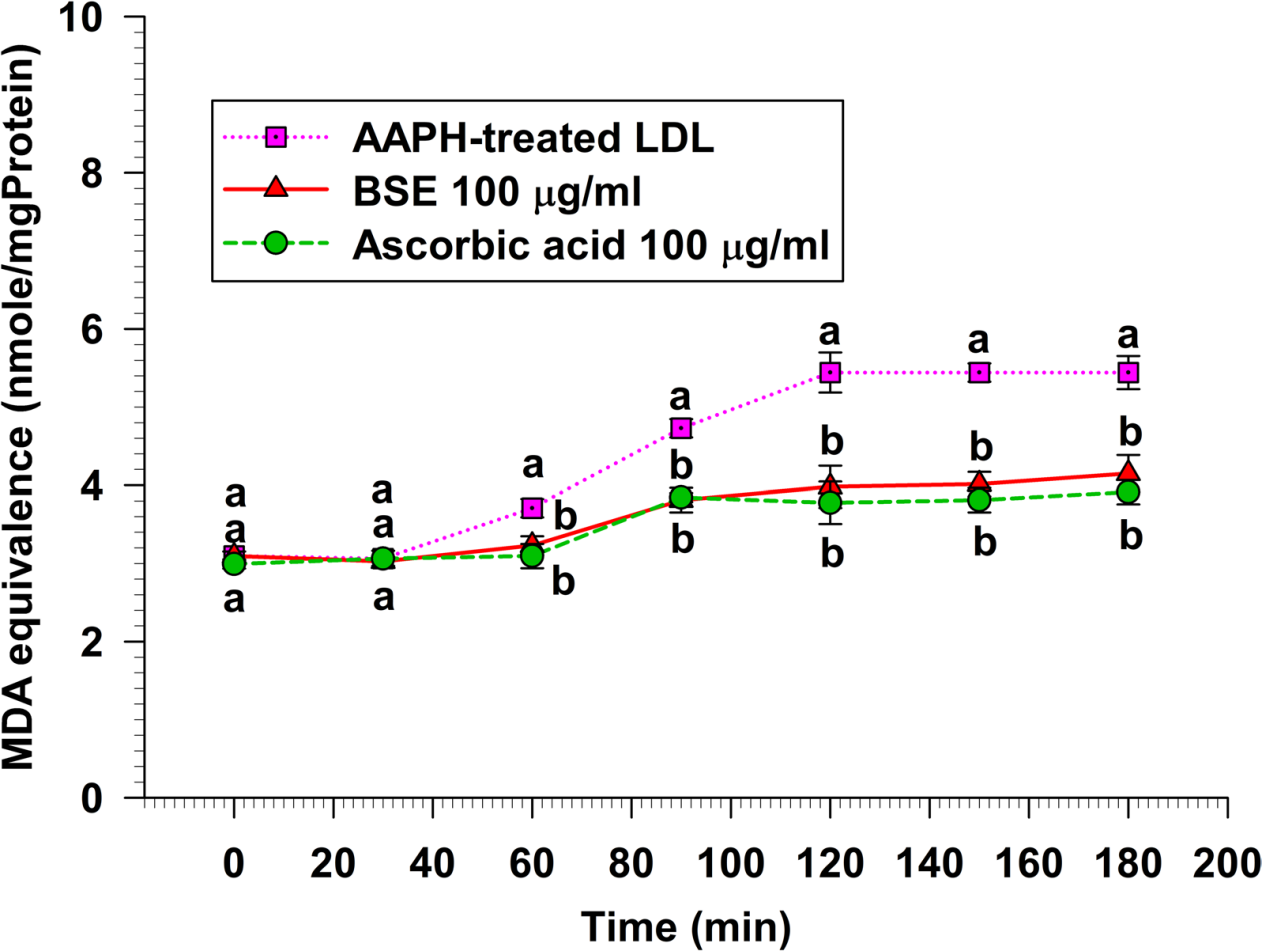
The AAPH-induced LDL oxidation scavenging activity

The LDL oxidation scavenging effect was investigated by AAPH-induced LDL oxidation assay. To induce LDL oxidation, isolated human LDL was challenged with AAPH, a potent ROS generator. For the experiment, BSE and ascorbic acid were used at 100 µg/ml concentrations. The samples were incubated for 3 h, and at various time points, aliquots were collected for analysis using the TBARS assay. The results are showed in Fig. 7 and Table 1. Both BSE and ascorbic acid exhibited a significant decrease in TBARS formation starting from 60 min of AAPH exposure until the end of the experiment at 3 h (*p* < 0.05). Based on these results, it can be inferred that both BSE and ascorbic acid had similar LDL oxidation scavenging properties.

Data represent the MDA equivalence at various times over 3 h. Each value represents mean ± SEM (*n* = 3) and is representative of three independent experiments with similar results. Means with the same superscript are not significantly different from each other (Tukey’s HSD test, *p* < 0.05).

### SIN-1-induced LDL oxidation activity

**Fig. 8:**
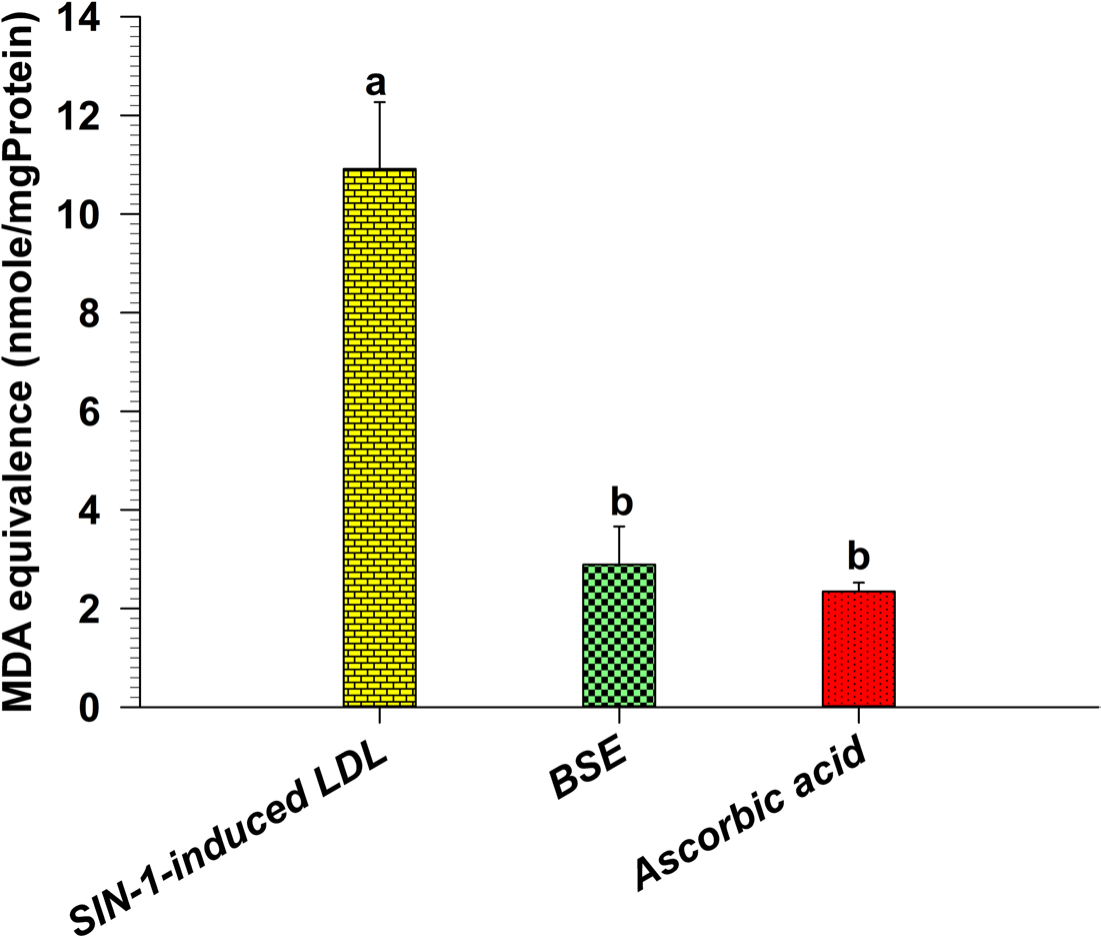
The SIN-1-induced LDL oxidation scavenging activity of BSE and ascorbic acid

In this study, SIN-1 is used as a generator of RNS to induce LDL oxidation. Isolated human LDL was incubated with SIN-1, BSE, and ascorbic acid for evaluation. Figure 8 and Table 1 illustrates the RNS scavenging effect of BSE and ascorbic acid at 100 µg/ml doses. Both BSE and ascorbic acid exhibited remarkably similar properties in combating the impact of RNS, as evidenced by their ability to reduce the concentration of MDA significantly.

Data represent the MDA equivalence at 18 h. Each value represents mean ± SEM (*n* = 3) and is representative of three independent experiments with similar results. Means with the same superscript are not significantly different from each other (Tukey’s HSD test, *p* < 0.05).

## Discussion

The present study was carried out to determine the antioxidant and anti-LDL oxidation activity in BSE compared with a naturally occurring organic compound with antioxidant properties. BSE is a well-known herb that has been traditionally used in Ayurvedic medicine, but its antioxidative properties have not been fully investigated. In this study, the antioxidative properties of BSE were investigated using DPPH, ABTS, FRAP, NO, and Peroxynitrite scavenging assay. Moreover, the study also examined the prevention effect of isolated human LDL oxidation by AAPH and SIN-1.

The BSE was examined as the main chemical constituent using LC–MS/MS technique. The results showed that genistein, daidzein, and biochanin A were found in BSE (Fig. 1). This result is in substantial agreement with previous findings that *B. superba* crude extract was isolated by column chromatography and PTLC, contained genistein, biochanin A, and daidzein [14, 25]. Moreover, BSE also found unidentified constituents that might synergistically affect antioxidant activity with three isoflavones identified.

The DPPH, ABTS, FRAP, NO, and Peroxynitrite scavenging capacity are important indexes to elucidate and compare the antioxidant activity of samples. The DPPH radical scavenging assay is widely used to measure the antioxidant potential of a compound, an extract, or other biological sources [26]. This assay determines that the free radical method is an antioxidant assay based on electron transfer that changes the color from the violet color of the DPPH radical to the yellow of the non-radical DPPH derivative at 515 nm. In this study, we evaluated the antioxidant activity of BSE. Remarkably, BSE exhibited potent antioxidant activity at all concentrations (Fig. 2). This result is consistent with Nuengchamnong et al. and [27] Sirichaiwetchakoon et al. [16] that an extract of *B. superba* has an antioxidative effect on the HPLC-DPPH method equipped with the MS/MS technique. Moreover, the antioxidant capacity of BSE was also evaluated using the ABTS^+^ radical cation decolorization assay. These findings indicate that BSE exhibits a notably superior ability to reduce ABTS^+^ (Fig. 3). There have been reported that the 5,7-dihydroxy structure of genistein and 4′-hydroxyl group in daidzein are responsible for the activity of isoflavones as scavengers of the radicals [28, 29]. The LC–MS/MS analysis revealed the presence of these compounds in BSE. So, the ABTS + scavenging activity of BSE may occur from genistein and daidzein. Furthermore, the FRAP assay is a widely used method for evaluating the antioxidant capacity of plant extract. The results obtained from the FRAP assay indicated that the ability of the samples could reduce ferric ions (Fe^3+^) to ferrous ions (Fe^2+^). In this investigation, our findings found that BSE had an antioxidant activity in a dose-dependent manner (Fig. 4). The genistein, daidzein, and biochanin A found in BSE might play an important role in this antioxidant activity. Several reports showed that these compounds had antioxidant activity using FRAP assay. The antioxidant ability of these isoflavones may occur from phenolic hydroxyl that are directly related to their antioxidant activity [30–34]. Nitric oxide is an essential signaling molecule involved in diverse physiological and pathophysiological, including neural signal transmission, immune response, vasodilation control, and blood pressure regulation [35, 36]. However, excessive NO can lead to inflammation, tissue toxicity, and the development of pathological conditions, particularly vascular diseases [37]. In this study, we investigated the nitric oxide scavenging capacity of BSE. Interestingly, the result presented that BSE exhibited significantly greater nitric oxide scavenging activity than gallic acid, a well-known potent antioxidant to scavenge NO (Fig. 5) [38, 39]. These findings provide evidence that BSE could have significant implications in preventing oxidative stress-related conditions and various pathologies associated with elevated NO levels. The excessive of peroxynitrite production, categorized as one of the RNS, plays a crucial role in the pathogenesis of various conditions, including alzheimer, parkinson, chronic heart failure, diabetes, cancer, and chronic inflammatory diseases [40–42]. The peroxynitrite scavenging assay of BSE expressed that BSE at a concentration of 62.5 µg/ml inhibited the bleaching effect of peroxynitrite on Evan Blue dye (Fig. 6). Besides, BSE exhibited the same peroxynitrite scavenging activity as gallic acid. The mechanism of peroxynitrite scavenging activity might occur by genistein and daidzein, which are found in BSE. Some studies have reported that isoflavones, genistein and daidzein could inhibit peroxynitrite oxidation [43–45]. These results lead us to believe that BSE has strong antioxidant property and could scavenge various reactive species, including DPPH, ABTS, FRAP, Nitric oxide, and Peroxynitrite.

LDL oxidation is pivotal in developing atherosclerotic vascular diseases [46]. Oxidative processes involving ROS and RNS are crucial mechanisms for LDL oxidation [47]. Lipoprotein oxidation induced in vitro in whole plasma is expected to represent a model of anti-LDL oxidation activity investigation in the body [48]. Therefore, we evaluated the anti-LDL oxidation activity of BSE using the TBARS assay with isolated human LDL challenged by AAPH as ROS generator and SIN-1 as RNS generator. AAPH can initiate lipid peroxidation and protein oxidation in isolated LDL particles. BSE demonstrated the anti-isolated human LDL oxidation by extending the lag time of AAPH-induced LDL oxidation assay compared to the control (Fig. 7). Moreover, it was found that BSE exhibited an equivalent level of anti-LDL oxidation to that of ascorbic acid, a potent anti-oxidant known to prevent low-density lipoprotein from oxidation [49]. The antioxidative effects of BSE against AAPH activity might be the effect of genistein, which is reported by Yoon et al. [50] that genistein could prevent LDL oxidation, radical scavenging action, activation of antioxidant enzymes, and suppression of oxidative DNA damage. Moreover, SIN-1, an RNS generator, is used to examine the anti-LDL oxidation. RNS, such as peroxynitrite, could induce oxidative stress within the human body. Besides, when LDL is oxidized by peroxynitrite, it exhibits a high affinity for scavenger receptors on macrophages, accumulating cholesteryl esters and contributing to the formation of fatty streaks and atherosclerotic lesions [51]. This study revealed that the RNS produced by SIN-1 was found to be scavenged by BSE (Fig. 8). Interestingly, BSE exhibited scavenger activity as potent as ascorbic acid. This finding is consistent with the peroxynitrite scavenging assay in this study. This is the first report that BSE could have an essential role in preventing isolated human LDL oxidation by inhibiting ROS and RNS-induced LDL oxidation technique.

## Conclusion

These findings provide evidence that BSE could exhibit free radical scavenging activities and anti-LDL oxidation effects in the in vitro model. The significance of these outcomes suggests a promising potential for BSE as a health supplement product with antioxidant properties, which may develop for preventing atherosclerosis and other diseases associated with oxidative stress. However, the limitation of this study is that it was performed in an in vitro model, so the findings might not be applicable to humans directly. Furthermore, the study might lack an in-depth exploration of the molecular mechanisms underlying the observed free radical scavenging and anti-LDL oxidation activities. Therefore, further studies are needed to be investigated, particularly the examinations of the mechanism of actions, pharmacokinetics, pharmacodynamics, and the determination of efficacious and safe dosages in humans.

## Data Availability

The datasets used or analyzed during the current study are available from the corresponding author upon reasonable request.

## Abbreviations

LDL: Low-density lipoproteins
DPPH: 2,2-Diphenyl-1-picrylhydrazyl
ABTS: 2,2-Azinobis-(3-ethylbenzothiazoline)-6-sulfonic acid
FRAP: Ferric Reducing Ability Power
NO: Nitric Oxide
AAPH: 2,2'-Azobis (2-amidinopropane) dihydrochloride
LC–MS/MS: Liquid Chromatography-Mass Spectrometer/Mass Spectrometer
SIN-1: 3-Morpholinosydnonimine hydrochloride
ROS: Reactive Oxygen Species
RNS: Reactive Nitrogen Species
BSE: *B. superba* Extract
NED: Naphthylethylenediamine Dihydrochloride
DTPA: Diethylene-triamine-pentaacetic acid
TCA: Trichloroacetic Acid
TPTZ: 2,4,6-Tris(2-pyridyl)-s-triazine
TEAC: Trolox Equivalent Antioxidant Capacity
TBARS: Thiobarbituric Acid Reacting Substances
MDA: Malondialdehyde
